# Sugar in the Urine of Cows in Paturient Apoplexia*Oesterreich. “Vierteljahrschift f. Veterinair Kunde.” Vol. 63.

**Published:** 1885-10

**Authors:** 


### SUGAR IN THE URINE OF COWS IN PARTURIENT APOPLEXIA.*

Nocord, of Alfort, repeatedly calls the attention of students in his lectures on obstetrics, to the similarity of the phenomena of this disease in cows to those of uræmia in man.

He has been much disappointed in the results of the chemical examination of the blood from such animals with regard to retained urates.

On the other hand, small quantities of albumen have been present in every case, and sugar in varying quantities. He has never been able to find the latter before in the examination of the urine of diseased animals.

Prof. Yvon, the chemist of the Alfort school, examined the urine from six cows having the disease, and found sugar in each, viz.: in one litre of urine, 1.10; 1.85; 6.60; 13.20; 25.30; 44.80 grammes, respectively. The first three animals recovered, the last three died from the disease. It is, therefore, probable that the percentage of sugar in the urine bears some relation upon the prognosis.

On recovery, the examination of the urine revealed the entire disappearance of the sugar. Sugar was never found in the urine of cows that had calved in a natural manner.

* Oesterreich. "Vierteljahrschift f. Veterinair Kunde." Vol. 63.

